# Heterogeneities in Cell Cycle Checkpoint Activation Following Doxorubicin Treatment Reveal Targetable Vulnerabilities in *TP53* Mutated Ultra High-Risk Neuroblastoma Cell Lines

**DOI:** 10.3390/ijms22073664

**Published:** 2021-04-01

**Authors:** Linnéa Ödborn Jönsson, Maryam Sahi, Ximena Lopez-Lorenzo, Faye Leilah Keller, Ourania N. Kostopoulou, Nikolas Herold, Lars Ährlund-Richter, Shahrzad Shirazi Fard

**Affiliations:** 1 Department of Women’s and Children’s Health, Karolinska Institutet, 171 64 Stockholm, Sweden; linnjons93@gmail.com (L.Ö.J.); msahi@kth.se (M.S.); ximena.lopez@scilifelab.se (X.L.-L.); faye.keller@gmx.de (F.L.K.); nikolas.herold@ki.se (N.H.); lars.ahrlund@ki.se (L.Ä.-R.); 2 Department of Oncology-Pathology, Karolinska Institutet, 171 64 Stockholm, Sweden; ourania.kostopoulou@ki.se; 3 Pediatric Oncology, Astrid Lindgren Children’s Hospital, Karolinska University Hospital Solna, 171 64 Stockholm, Sweden

**Keywords:** ATM, cell cycle arrest, DNA damage, drug-resistance, high-risk neuroblastoma, mitotic-arrest, p21, proliferation

## Abstract

Most chemotherapeutics target DNA integrity and thereby trigger tumour cell death through activation of DNA damage responses that are tightly coupled to the cell cycle. Disturbances in cell cycle regulation can therefore lead to treatment resistance. Here, a comprehensive analysis of cell cycle checkpoint activation following doxorubicin (doxo) treatment was performed using flow cytometry, immunofluorescence and live-cell imaging in a panel of *TP53* mutated ultra high-risk neuroblastoma (NB) cell lines, SK-N-DZ, Kelly, SK-N-AS, SK-N-FI, and BE(2)-C. Following treatment, a dose-dependent accumulation in either S- and/or G2/M-phase was observed. This coincided with a heterogeneous increase of cell cycle checkpoint proteins, i.e., phos-ATM, phos-CHK1, phos-CHK2, Wee1, p21^Cip1/Waf1^, and p27^Kip^ among the cell lines. Combination treatment with doxo and a small-molecule inhibitor of ATM showed a delay in regrowth in SK-N-DZ, of CHK1 in BE(2)-C, of Wee1 in SK-N-FI and BE(2)-C, and of p21 in Kelly and BE(2)-C. Further investigation revealed, in all tested cell lines, a subset of cells arrested in mitosis, indicating independence on the intra-S- and/or G2/M-checkpoints. Taken together, we mapped distinct cell cycle checkpoints in ultra high-risk NB cell lines and identified checkpoint dependent and independent druggable targets.

## 1. Introduction

Neuroblastoma (NB) is the most common extracranial solid tumor in early childhood. It is a heterogenous neoplasia with clinical presentation ranging from spontaneous regression of metastatic disease to a rapidly progressive course [1]. Fifty percent of all children diagnosed with NB are considered to be high-risk. Among high-risk NB, a subset of approximately 19% is considered to be ultra-high risk, defined as death from disease within 18 months of diagnosis despite intensive treatment [2]. Furthermore, relapse rates in high-risk NB are as high as 50% despite intensive multimodal treatment and are frequently characterised by therapy resistance and intra-tumor diversity making it difficult to cure [3,4]. Ultra high-risk NB and relapsed NB remain a therapeutic challenge with only limited evidence for efficacious salvage therapies [5].

Small-molecule inhibitors of anaplastic lymphoma kinase (ALK), antibodies against the NB antigen GD2 and radiolabelled somatostatin analogues [6,7] represent novel targeted strategies, but chemotherapy (CT) continues to be the cornerstone in systemic NB treatment. The anthracyclin doxorubicin (doxo) is included in a wide variety of cancer treatment protocols for children and adults, and is an established salvage therapy if anthracycline-free induction CT is not successful [8]. Doxo exerts its function through DNA intercalation, induction of DNA strand breaks, direct inhibition of topoisomerase II and free radical formation, the most substantial effect being the induction of DNA damage [9].

DNA damage leads to the activation of cell cycle checkpoint surveillance mechanisms causing cells to either arrest in order to ensure DNA repair [10] or to undergo cell death, if the DNA damage is unrepairable [11]. These checkpoints involve signaling pathways governed by various kinases, namely, the ataxia telangiectasia mutated serine/threonine kinase (ATM) and the checkpoint kinases 1 and 2 (CHK1/2) [12]. The CHK1/2 kinases exert indirect checkpoint control through several downstream targets, including Wee1 and p21^Cip1/Waf1^ (p21), which in turn inhibit the catalytic activity of cyclin-dependent kinases [13,14]. Therefore, activation of the cell cycle checkpoint proteins consequently causes cell cycle arrest [15]. Furthermore, while p21 is mainly upregulated during acute arrest, upregulation of the Cip/Kip family member p27Kip1 maintains a prolonged arrest following DNA damage [16].

While checkpoint proteins maintain genomic intregitry, dysregulation might be associated with therapy resistance [17]. Among common genetic alterations, 50% of high-risk NB have an amplification of the MYCN oncogene, which is able to transcriptionally upregulate checkpoint proteins including CHK1 following treatment. This indicates that these subsets of high-risk NB cells might benefit from treatments targeting CHK1, thereby making it an ideal target for therapeutic intervention [1,18,19]. On the contrary many checkpoint proteins, including ATM and CHK1, are encoded on 11q, which is commonly lost in high-risk NB, leading to reduced expression of these proteins [20]. Instead, the corresponding signaling pathways may be compensated for by the function of another compensatory checkpoint. Furthermore, whereas alterations in either MYCN or 11q are commonly present at diagnosis, *TP53* mutations (mut) at diagnosis are rare, occurring in <2% of cases independent of tumour stage [21]. Instead, abnormalities in the p53 pathway, including *TP53* mut, are identified in 49% of NB following relapse, indicating a mechanistic relevance in the development of therapy resistance [22]. In addition, abnormality in the p53 pathway results in a deficient G1/S-checkpoint, necessitating reliance upon the intra-S- and/or G2/M-checkpoint for continued survival [23]. Therefore, the potential of small molecular inhibitors targeting the intra-S- and/or G2/M-checkpoint, either as single-agents or in combination with CT, are of interest, especially in *TP53* mut NB.

Here, we utilized a panel of five ultra high-risk *TP53* mut NB cell lines, SK-N-DZ, Kelly, SK-N-AS, SK-N-FI, and BE(2)-C, to study cell cycle checkpoints and regrowth capacity following doxo treatment. The aim is to increase the understanding of conditions leading to treatment resistance and relapse, and to identify targetable vulnerabilities. 

We demonstrate heterogeneity in cell cycle distribution among the tested cell lines following doxo treatment. Furthermore, a fraction of cells showed activation of checkpoint proteins and sensitivity to chemical inhibition of these proteins. However, a subset of cells were able to progress though the S-phase and arrest in mitosis, indicating independence of the intra-S- and G2/M-checkpoints. These results demonstrate heterogeneity in cell cycle regulation with the presence of both checkpoint-dependent and independent subpopulations of tumour cells in all tested cell lines. Identification of CT escape mechanisms does not only increase the knowledge on chemoresistance–but helps to identify specific vulnerabilities that can be targeted in conjunction with conventional therapy.

## 2. Results

### 2.1. Assessments of Cytotoxic Effects Following Doxo Treatment

The cytotoxic effect of doxo has been shown to be highly varying among p53 inactive NB cell lines, with IC50 values ranging from 0.06 to 0.2 µM [24]. To investigate resistance to doxo-induced apoptosis among our panel of *TP53* mut cell lines, we measured DNA fragmentation (sub-G1) after 48 h of treatment with doxo serially diluted at 0.01 µM, 0.1 µM and 1 µM, using flow cytometry. Importantly, the highest dose tested, 1 µM, corresponds to clinically relevant plasma peak level in patients [25,26].

**Figure 1:**
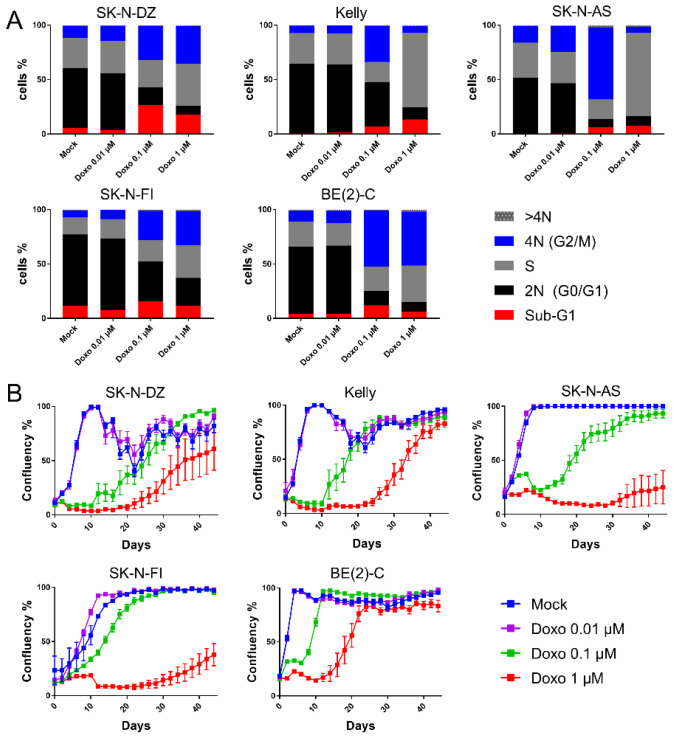
Analysis of cell cycle distribution and regrowth in neuroblastoma (NB) cell lines following treatments with indicated doses of mock (PBS) or doxo. (**A**) HOECHST 33343 flow cytometry was performed on SK-N-DZ, Kelly, SK-N-AS, SK-N-FI, and BE(2)-C following mock treatment (PBS) or titrations of doxo at indicated concentrations. Samples were analysed 48 h post-treatment. Results are presented as percentage cells in each cell cycle phase. The lowest tested dose gave similar results as the mock-treatment, indicating 0.01 µM as a suboptimal dose. Treatment with either 0.1 µM or 1 µM resulted in a dose-dependent reduction in G0/G1- together with accumulation in S- and/or G2/M-phases for all tested cell lines. Mean of three experiments. (**B**) Cultured cells were exposed to the indicated treatment (doxo/mock) and, analysed using live-cell imaging using the IncuCyte at the indicated time points. The confluence shows the cellular densities of each cell line measured over 44 days. Two cell lines, SK-N-DZ and Kelly, showed in the mock-treated samples detachment from the plates once reaching high confluency, this was most likely due to crowding effects. The lowest tested dose followed the same growth pattern as with mock treatment. Following either 0.1 µM or 1 µM all cell lines showed regrowth capacity, albeit after an extended lag period. Mean ± SEM of five experiments.

As shown in Table 1 and in Figure 1A, a significant increase in the sub-G1 fractions were detected for SK-N-DZ cultures after 0.1 µM and 1 µM doxo as compared to control (26% and 18% vs. 5.3%). Kelly and SK-N-AS presented increased sub-G1 fractions after 1 µM doxo (from 0.9 to 13%, *p* < 0.01 and from 0.5 to 7.4%, *p* < 0.05, respectively). However, SK-N-FI and BE(2)-C showed no change in sub-G1 fractions after any of the tested concentrations. Thus, the data indicated a high resistance to doxo-induced apoptosis, except in SK-N-DZ, Kelly, and SK-N-AS cell lines, where full resistance was not observed.

### 2.2. Cell Cycle Distribution and Regrowth Dynamics Following Doxo Treatment

Since doxo-induced DNA damage is suggested to trigger cell cycle arrest, we investigated cell cycle distribution following doxo exposure using flow cytometry. Forty-eight hours following treatment with the lowest tested dose (0.01 µM doxo), all cell lines maintained the bulk of cells in G0/G1 (46–66%), similar to the results after mock-treatment (51–66%).

Treatment with the intermediate dosing (0.1 µM doxo), resulted in accumulation of G2/M-phases in all cell lines as compared to mock treatment (27–66% vs. 7–15%; *p* < 0.0001), with a decreased G0/G1 population (7–41% vs. 46–66%; *p* < 0.0001) Figure 1A and Table S3. Furthermore, for Kelly and SK-N-AS, the S-phase population was reduced, which was not observed for SK-N-DZ, SK-N-FI, and BE(2)-C Figure 1A and Table S3.

The highest tested dose (1 µM doxo) presented, similar to 0.1 µM, decreased levels of diploid cells (G0/G1) in all cell lines. Notably, all cell lines, except BE(2)-C, presented S-phase accumulation, suggesting an intra-S-phase arrest. Especially two cell lines, Kelly and SK-N-AS, showed a high preference for S-phase accumulation reaching levels of approximately 70% compared to 30% when mock-treated Figure 1A and Table S3. Furthermore, while Kelly and SK-N-AS presented only a minor fraction of the cells in the G2/M-phase (6 and 5%, respectively), the remaining three cell lines, SK-N-DZ, SK-N-FI, and BE(2)-C, showed concomitant increase in G2/M-phase, indicating a p53-independent G2/M-phase arrest Figure 1A and Table S3.

To determine the long-term effect of doxo treatment, regrowth assays were conducted. Regrowth (confluency) was mapped during a period of 44 days following a pulse treatment of mock or doxo at 0.01 µM, 0.1 µM or 1 µM, for 48 h. Two cell lines, SK-N-DZ and Kelly, showed detachment from the plates once reaching high confluency following mock treatment, most likely due to crowding effects. Following the lowest tested dose (0.01 µM doxo) regrowth followed the same growth pattern as with mock treatment. Moreover, for all cell lines, except BE(2)-C, a dose dependency with a lag period of approximately 20 days following 0.1 µM doxo, and 24 days following 1 µM doxo was observed before an increase in confluency (regrowth). However, BE(2)-C cells showed regrowth at day 6 following 0.1 µM and at day 14 following 1 µM doxo, indicating a less prominent lag period compared to the other four cell lines Figure 1B. Importantly, the duration of the lag period prior to regrowth following doxo treatment did not directly reflect drug sensitivity Table 1 but seems largely influenced by the intrinsic proliferation rate of each cell line with BE(2)-C having the shortest doubling time, followed by SK-N-DZ, Kelly, SK-N-AS, and SK-N-FI.

The combined data illustrate heterogeneity among the *TP53* mut cell line in their cell cycle response upon doxo treatment. This is especially seen after 1 µM doxo treatment, where SK-N-AS and Kelly accumulated in S-phase, SK-N-DZ and SK-N-FI in both S- and G2/M-phase and BE(2)-C in G2/M-phase. Furthermore, all cell lines showed regrowth capacity following all tested concentrations of doxo, albeit with different kinetics indicating the presence of resistant cells.

### 2.3. Assessment of Cell Cycle Checkpoint Activation Following Doxo Treatment

Next, the activation of checkpoint proteins was investigated following doxo treatment by determining the expression of ATM-phospho S1981 (pATM), CHK1-phospho S296 (pCHK1), CHK2-phospho T68 (pCHK2), Wee1, p21, and p27^Kip1^ in single-cells using immunofluorescence. This was done to correlate doxo-induced alterations in the cell cycle distribution with intra-S- and/or G2/M-checkpoint activation.

**Figure 2:**
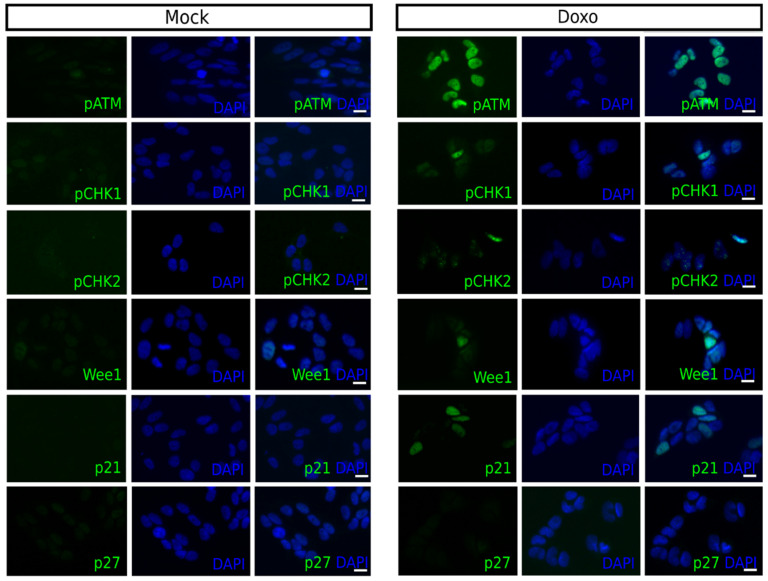
Expression of cell cycle checkpoint proteins in BE(2)-C cells after doxo treatment. Cultured cells were exposed to the indicated treatment (doxo/mock) and analysed for expression of pATM, pCHK1, pCHK2, Wee1, p21, and p27 after 48 h. Increased expression of all markers, except p27, was observed in the BE(2)-C cell line following treatment. pATM showed a high increase in expression (86% positive cells), whereas the pCHK1, pCHK2, Wee1, and p21 markers were detected at a low frequency (between 6–21% positive cells). Scale bar = 20 µm.

Forty-eight hours following 1 µM doxo treatment a robust increase in the number of pATM positive cells was observed for all cell lines, except SK-N-AS. The fraction of positive cells increased for pCHK1 in Kelly and BE(2)-C, and for pCHK2, a downstream target of ATM, for all cell lines, except SK-N-AS Table 2. The fraction of Wee1 positive cells increased in SK-N-DZ, Kelly, and BE(2)-C, while the fraction of p21 positive cells increased for all cell lines, except Kelly. However, Kelly and SK-N-FI had a high fraction of p21 positive cells, approximately 20%, following mock treatment. Both of these cell lines also showed an increase in the fraction of p27^Kip1^ positive cells following treatment Table 3. Overall, the BE(2)-C cell line showed the highest increase in the fraction of cells positive for cell cycle checkpoint proteins following 1 µM doxo treatment and representative pictures of each marker are presented in Figure 2.

These results illustrate that the doxo-induced frequencies of cells positive for cell cycle checkpoint proteins, pCHK1, pCHK2, Wee1, p21 and p27^Kip1^ were generally low, the exception was pATM which was strongly increased for all cell lines, except SK-N-AS.

### 2.4. Sensitivity to Inhibitors of Cell Cycle Checkpoint Proteins in Combination with Doxo Treatment

An increase in the number of positive cells for one or several cell cycle checkpoint proteins was shown after doxo treatment for all NB cell lines, indicating their role in doxo-induced cell cycle arrest.

To validate the functional role of cell cycle checkpoint proteins we employed pharmacological inhibitors. Small molecular inhibitors of ATMi (KU 60019), CHK1i (PF-477736), CHK1/2i (AZD 7762), Wee1i (Adavosertib), and p21i (UC2288) were tested in order to assess the effect of the inhibitors on NB viability and growth. A small molecular inhibitor was not commercially available for p27^Kip1^.

**Figure 3:**
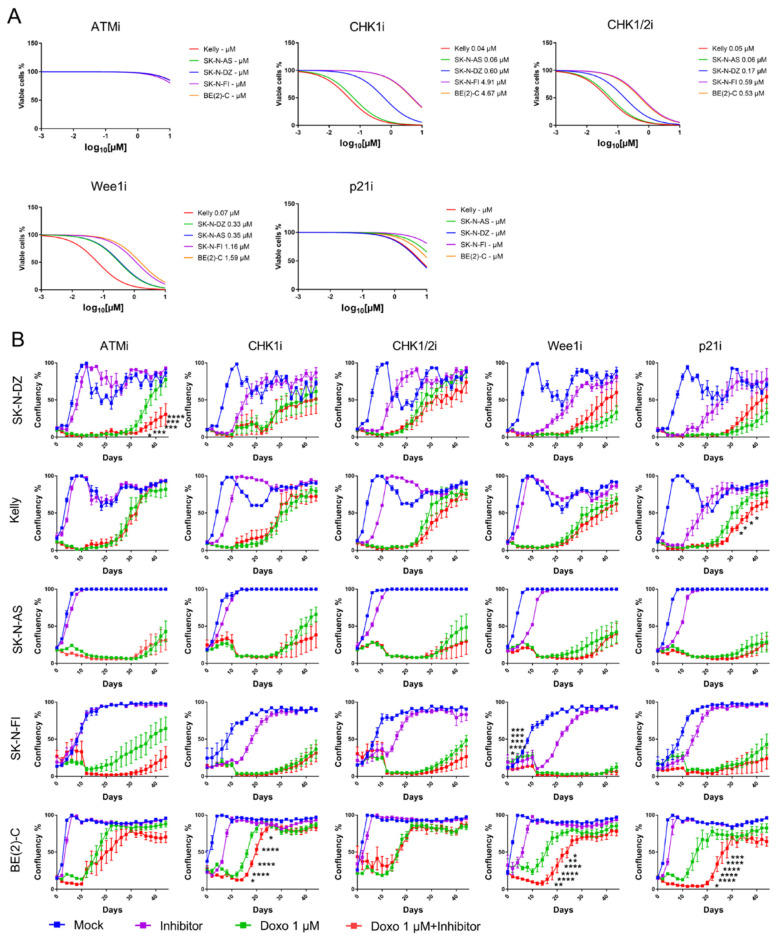
Cell viability assay and long-term cell growth (confluency) assay on NB cell lines following treatments with inhibitors of the cell cycle checkpoint proteins. NB cell lines were treated with chemical inhibitors of ATM, CHK1, CHK1/2, Wee1, or p21. (**A**) IC50 values were calculated using an MTS assay 72 h post-treatment as the concentration of drug necessary to inhibit 50% cell viability compared to mock-treated cells. None of the cell lines showed sensitivity for ATMi or p21i. Kelly and SK-N-AS showed the highest sensitivity to CHK1i, CHK1/2i, and Wee1i, followed by SK-N-DZ, SK-N-FI and BE(2)-C. N = 3. (**B**) NB cell lines were treated with inhibitors of ATMi, CHK1i, CHK1/2i, Wee1i, or p21i. Regrowth was measured over 44 days with an IncuCyte (“confluence” value indicates the cellular densities). All cell lines, except SK-N-AS, showed a reduction in growth following combination treatment with doxo compared to a single doxo treatment. BE(2)-C showed the highest sensitivity for the inhibitors. Mean ± SD of five experiments. * = *p* < 0.05, ** = *p* < 0.01, *** = *p* < 0.001, **** = *p* < 0.0001, *NS* = not significant *p* > 0.05. Student’s *t*-test. Two-way ANOVA with Bonferroni post-tests.

The ability of each drug was tested as a single-agent, using a 4-log dose–response cell viability assay with a 72-h time point. Inhibition of ATM or p21 did not give a decrease in cell viability post-treatment for any of the cell lines. However, all tested NB cell lines were sensitive to single-agent inhibitions of CHK1, CHK1/2, or Wee1, compared to mock treatment. Kelly and SK-N-AS showed the most sensitivity, followed by SK-N-DZ, SK-N-FI, and BE(2)-C Figure 3A.

Current developmental strategies for inhibitors of cell cycle checkpoint proteins have been focused on their chemosensitizing properties. Here, a sub-sequential assessment of the ability of the molecular inhibitors to potentiate the CT doxo in the panel of NB cell lines was made. Each inhibitor was combined at a concentration of 10 µM for ATMi and p21i or the corresponding IC50 value with 1 µM doxo and cellular growth (confluency) was measured for 44 days.

A significant delay in growth was observed in the SK-N-DZ cell line after combination treatment with doxo and ATMi compared to single doxo treatment (*p* < 0.05–0.0001). Kelly showed delay in growth following combination treatment with doxo and p21i (*p* < 0.05) and BE(2)-C showed delay in growth following combination treatment with doxo and either CHK1i (*p* < 0.05–0.0001), Wee1i (*p* < 0.05–0.0001), or p21i (*p* < 0.05–0.0001). SK-N-FI showed an initial delay in growth (between day 2–6, *p* < 0.05–0.0001) following combination treatment with doxo and Wee1i however, this was not observed from experimental day 8 and later. None of the combination treatments showed a delay in growth compared with a single doxo treatment in the SK-N-AS cell line Figure 3B. Overall, these results demonstrate that all cell lines, except SK-N-AS, delayed regrowth when doxo was combined with an inhibitor of the cell cycle checkpoint proteins suggesting that the inhibitors potentiate doxo in the suppression of growth.

### 2.5. Presence of Mitotic Arrest Following Doxo Treatment

The data indicate that there is a high heterogeneity among the different cell lines, both in regard to their expression of cell cycle checkpoint proteins but also in their sensitivity to chemical inhibitors of the checkpoint proteins. Furthermore, combination treatment was not able to abolish regrowth, rather only delay it in some cell lines. We hypothesized that there might be an additional subset of cells, following doxo treatment, which is independent of the intra-S- and/or G2/M-checkpoints. The presence of mitotic arrest following doxo treatment was therefore investigated. Using immunofluorescence, cells were stained for the expression of PH3, specifically expressed in the late G2/M-phase with the highest levels during M-phase [27].

After doxo treatment, an increase in the fraction of PH3 positive cells (indicating mitotic arrest) was observed in all cell lines, except for Kelly. SK-N-FI and BE(2)-C displayed the highest fraction of cells in mitotic arrest following doxo treatment reaching 20% and 15% PH3 positive cells respectively Table 4. Furthermore, cell cycle behaviour, prior to doxo-induced mitotic arrest, was also investigated by co-staining with PH3 and the thymidine analogue EdU, indicating active replication /S-phase progression during the treatment.

A significant reduction of EdU positive cells was observed in all tested cell lines after exposure to 1 µM doxo, as expected from doxo-induced cell cycle arrest. The most dramatic reductions of EdU positive cells, indicating abundant arrest, were noted for Kelly and SK-N-FI (from 92 to 20% and 65 to 27%, respectively). Despite statistically significant reductions, SK-N-DZ, SK-N-AS, and BE(2)-C, maintained relatively high levels, ranging 64–81%, of EdU positive cells following doxo treatment Table 4.

**Figure 4:**
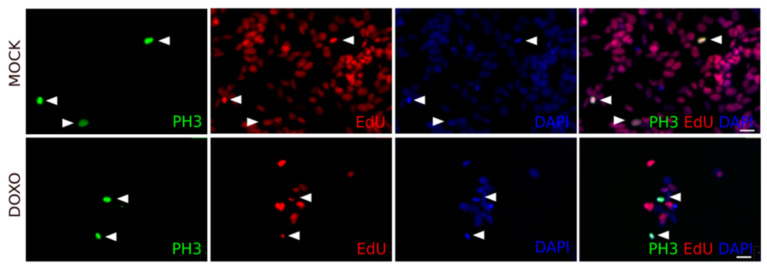
Cell cycle progression in BE(2)-C cells following doxo treatment. BE(2)-C cells treated with either mock or doxo (1 µM). PH3+/EdU+ cells were present both in the mock and doxo treatment cultures. Arrowhead: PH3+/EdU+ cells. Scale bar 50 µm.

A small, but significant, increase in PH3+/EdU+ cells (between 4.7–9.3%) was detected in SK-N-DZ, SK-N-AS, SK-N-FI, and BE(2)-C, but not in Kelly, which showed a reduction to 0.8% PH3+/EdU+ cells Table 4 and Figure 4. This indicated that a subset of cells were able to bypass the intra-S- and G2/M-checkpoints followed by an arrest in mitosis.

Furthermore, all tested cell lines showed an increase in PH3+/EdU- cells Table 4, indicating mitotic arrest without prior progression through the S-phase.

Considering the longstanding clinical experience of enhanced anti-tumour effect from repeated CT we investigated cell cycle behaviour after repeated doxo treatment, were 1 µM doxo was added for 48 h followed by medium change and a second dose of 1 µM doxo in combination with EdU for another 48 h.

In all tested cell lines, after adding doxo (1 + 1 µM), resilient cells could be shown to mostly reside in cellular arrest, as demonstrated by either increased expression of PH3 and/or failure to incorporate EdU Table 5. Nonetheless, the relative proportions of arrested cells differed among the cell lines, with Kelly, SK-N-FI, and BE(2)-C showing the highest fraction of cells in mitotic arrest and the lowest fraction of EdU positive cells.

Furthermore, a small fraction of PH3+/EdU+ cells (0.8–3.5%) were detected in all cell lines, except Kelly Table 5, indicating mitotic arrest with prior progression through the S-phase during the second doxo treatment. All tested cell lines also showed an increase in PH3+/EdU- cells Table 5, indicating mitotic arrest without prior progression through the S-phase.

These data illustrate the presence of cells in mitotic arrest (PH3 positive) following both single and repeated doxo treatment in all tested cell lines. Moreover, the subset of cells arrested in mitosis can be further subdivided depending on their cell cycle behavior, indicated by cells either with or without progression through an S-phase prior to the arrest.

### 2.6. Further Studies of Recurrence and Colony Formation Following Doxo Treatment

To investigate the consequence of replicating cells during treatment, we pulse labelled with EdU during the single 1 µM doxo treatment and examined regrowth capacity (colonies). Three cell lines, Kelly, SK-N-FI, and BE(2)-C, were selected as they displayed a range of cell cycle behaviours following doxo treatment, with Kelly preferentially accumulating in S-phase and having a low fraction of EdU positive cells (20%), whereas SK-N-FI showed accumulation in both S- and G2/M-phase and had a low fraction of EdU positive cells (27%) and BE(2)-C showed accumulation in G2/M-phase and had a higher fraction of EdU positive cells (64%) Table 4 and Table S3.

**Figure 5:**
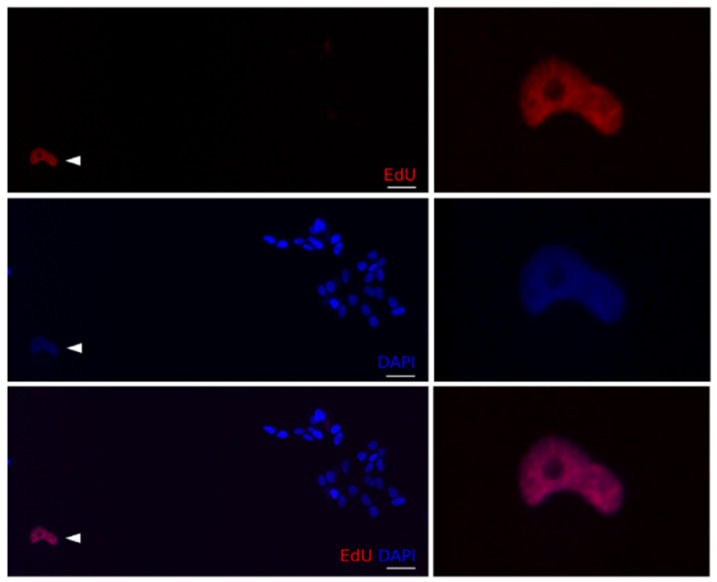
EdU positive BE(2)-C cells present at regrowth following single doxo treatment. BE(2)-C cells treated doxo (1 µM) in combination with EdU. A threshold of >10 cells in proximity was set for the definition of a colony, located on the right. EdU positive cells were present not directly adjacent to a regrowth colony. Arrowhead: EdU positive cell. Magnification of EdU positive cell shown on the left. Scale bar 50 µm.

In the Kelly cell line, EdU positive cells persisted in 2/45 newly formed colonies, in SK-N-FI EdU positive cells persisted in 12/36 newly formed colonies and in BE(2)-C EdU positive cells persisted in 37/40 newly formed colonies. Besides observing EdU positive cells in colonies, there was also a fraction of EdU positive cells not directly adjacent to colonies Figure 5. These cells counted for, on average per coverglas, 5 ± 4 EdU positive cells for Kelly, 17 ± 8 EdU positive cells for SK-N-FI and 23 ± 9 EdU positive cells for BE(2)-C. The number of remaining EdU positive cells was lower in Kelly compared to BE(2)-C (*p* < 0.05, N = 3, Student’s *t*-test) but not compared to SK-N-FI (*p* > 0.05, N = 3, Student’s *t*-test). The consequence of these EdU positive cells remains to be investigated.

Taken together, the results indicate heterogeneity among the NB cell lines regarding the fraction of regrowth colonies with descendants from tumor cells completing replication during the doxo challenge.

Based on our findings, and due to having the highest fraction of EdU positive colonies, the BE(2)-C cell line was selected for additional regrowth tracing using EdU in combination with repeated (1 + 1 µM) doxo treatment. BE(2)-C cells were pulse labelled with EdU during the second dose of doxo and colonies were investigated for the presence of EdU positive cells. However, no EdU positive cells were detected in 63/63 identified colonies. This suggests that cells responsible for regrowth following repeated doxo treatments have not progressed through an S-phase, at least during the second doxo dose. 

In fact, investigating the fraction of BE(2)-C cells positive for cell cycle checkpoint proteins following repeated doxo Table S4 showed a high fraction of pATM positive cells (1.3 to 90%, *p* < 0.001), similar to that found following a single doxo treatment. The fraction of pCHK1 or pCHK2 positive cells was reduced, reaching levels similar to the mock treatment. However, there was a persistent increase in the fraction of cells positive for Wee1 (8.4 to 17%; *p* < 0.01), and p21 (1.7 to 39%; *p* < 0.001), supporting the notion of sustained checkpoint activation. Furthermore, there was also an increase in the fraction of p27^Kip1^ expressing cells (0.0 to 2.8%, *p* < 0.01) indicating that a subset of cells was arrested for an extended period of time.

These data illustrate that cell progression through the S-phase during single doxo treatment is able to regrow. However, this is not the case when doxo is given repeatedly. Instead, cells that are most likely already arrested following the first doxo treatment, are capable of regrowth.

## 3. Discussion

The challenge of ultra high-risk and relapse tumours remains a major hurdle in the successful treatment of NB. Cell cycle checkpoint activation following therapy has been linked to tumour cell survival and although exploiting these checkpoints has been a successful treatment strategy in several adult tumours [28], the potential of their use in the treatment of pediatric tumours has only recently been explored.

Here, the cell cycle checkpoint activation following treatments with doxo was investigated in a panel of five in vitro ultra high-risk *TP53* mut NB cell lines. The flow cytometry data showed, for all cell lines, high, but not full, resistance to doxo induced apoptosis Table 1, in agreement with previous literature and a proposed role of p53 in the induction of cell death [11]. Furthermore, a dose-dependent decrease of cells in G0/G1 (2N-fraction) and an accumulation in S- and/or G2/M-phase was observed in all cell lines following either 0.1 or 1 µM doxo, in agreement with the notion that cells with a dysfunctional p53 pathway generally fail to arrest at the G1/S-checkpoint [29,30]. However, the cell response was dose-dependent, where for example the two cell lines, SK-N-AS and Kelly, which showed preferred accumulation in S-phase following 1 µM doxo, instead had preferred accumulation in G2/M-phase following 0.1 µM doxo. This indicates that the response is dependent on the extent of the DNA damage. These results corroborate the effect of different drug concentrations on cell cycle checkpoints, especially since tumour cells can experience non-lethal doses due to unavoidable rapid decline in drug concentration [9], hence creating several different CT resistant subpopulations which need to be targeted differently.

Among common genetic alterations in NB, *MYCN* amplification contributes to driving proliferation but it also transcriptionally upregulates CHK1 [31]. Three of the tested cell lines, SK-N-DZ, Kelly, and BE(2)-C, harbour an *MYCN* amplification, and two of these upregulated CHK1 following doxo treatment, whereas only BE(2)-C showed a reduction in growth following combination treatment with CHK1i. Moreover, BE(2)-C cells also responded with delay in growth following combination treatment with Wee1i and p21i, in agreement with an active G2/M-checkpoint. On the other hand, SK-N-DZ, which following doxo treatment displayed the highest fraction of pATM expressing cells (89%) among the tested cell lines, showed a delay in growth following combination treatment with ATMi, indicating its sensitivity. Synthetic lethality has been suggested between inhibitors of CHK1 and ATM, thus this combination might be an alternative in *MYCN*-amplified NB cells [32].

Moreover, the SK-N-AS cell line, which harbours the loss of one 11q copy rendering the cell line with impaired functional ATM and CHK1 due to haploinsufficiency [33], showed an increase of p21-expressing cells following doxo treatment. In fact, p21 has been shown to act as a suppressor of replication in a coordinated regulatory network that is orchestrated by Poly ADP-ribose polymerase 1 (PARP1) [34]. Moreover, PARP is able to monitor replication forks following DNA damage during S-phase, thereby facilitating intra-S-phase checkpoint activation [35]. This is in line with our results showing a preference for S-phase accumulation following doxo treatment in the SK-N-AS cell line Figure 1A. Since none of the tested combination treatments with cell cycle checkpoint inhibitors and doxo delayed regrowth in SK-N-AS, the addition of PARP inhibitors, either alone or in combination with inhibitors of p21, to second-line CT in this risk group should be explored further [36].

Moreover, when using a single-cell approach we were able to detect heterogeneity in the endogenous expression of cell cycle checkpoint proteins among the different NB cell lines. Both Kelly and SK-N-FI displayed a high fraction of p21 expressing cells (18% and 23%, respectively) in the mock-treated samples, both also showed increased S-phase accumulation and few EdU positive cells (20% and 27%, respectively) following doxo treatment. This cell cycle behaviour might be correlated to a p53 independent expression of p21 since endogenous expression of p21 has been linked to cell cycle behaviour with high expression leading to a senescence fate [37]. Furthermore, Kelly showed a delay in growth following co-inhibition with p21i and doxo, likely due to the endogenous high p21 expression, indicating a role of p21 in regulating the doxo response. The SK-N-FI cell line only showed an initial reduction in growth, which was not maintained, following combination treatment with Wee1i. 

Our results, even though they illustrate a heterogeneic response among NB cell lines summarized in Table S5, are in line with other studies where combination treatment with inhibitors of the cell cycle checkpoint proteins and CT sensitizes tumour cells resulting in reduction, but not abolishment, of cellular growth [32,38,39]. This further suggests the presence of a subset of arrested cells that are sensitive to chemical inhibitors of the intra-S- and/or G2/M-checkpoint proteins. However, many clinical trials targeting the checkpoint proteins have been terminated due to toxicity and/or low target specificity [28]. One explanation could be that the DNA damage response is a highly orchestrated network that might render the cells relatively insensitive to the antiproliferative effect of a single cell cycle checkpoint protein inhibitor [40]. Therefore it has been proposed to use a combination of these agents in order to enhance the efficiency of conventional CT [28]. Nevertheless, the contribution of additional doxo-resistant subpopulations cannot be excluded and therefore we suggest an alternative, or complementary, interpretation proposing the existence of resistant tumor cells not dependent on the intra-S- and/or G2/M-checkpoints. Labeling with the mitotic marker PH3 revealed an increase in PH3 positive cells (mitotic arrest) for all cell lines following either a single or a repeated doxo treatment Table 4 and Table 5. Recent studies have provided evidence for the importance of mitotoic arrest in both the formation and treatment of tumours, where abnormalities in the mitotic process are able to contribute to increased aneuploidy, polyploidy and structural DNA damage [41].

Furthermore, co-labeling with PH3 and EdU revealed that a subset of cells in mitotic arrest had progressed through the S-phase during the single doxo treatment. These cells seem to have bypassed the intra-S- and the G2/M-checkpoint, a phenomenon known as checkpoint adaptation [42]. Although most cells that undergo checkpoint adaptation die, a small number of cells have been shown to survive [43,44]. Our data revealed that a fraction of newly formed colonies in Kelly (4%), SK-N-FI (33%), and BE(2)-C (92%) included descendants from cells actively replicating during the 48 h window of doxo treatment, i.e., cells passing the S-phase, indicating the cellular source of regrowth. However, further studies are needed to clarify the proportions of actively replicating (EdU positive) cells that might undergo checkpoint adaptation following treatment. Interestingly, it has been suggested that ATM synergises with p21 to inhibit Cdk2 activity in checkpoint-adapting cells, which are then programmed to enter a state of quiescence [45]. Our results are in line with this phenomenon since there was a decrease in replicating (EdU positive) cells and an increase in the fraction of cells positive for pATM, p21, and p27^Kip1^ in the BE(2)-C cell line following repeated doxo treatment, indicating maintained arrest and/or quiescence.

The presence of a subset of resistant cells that are able to actively replicate during the treatment adds complexity when it comes to sensitizing tumour cells to DNA damaging drugs. One alternative treatment comes from a study where a CHK1/2 inhibitor revealed strong synergy with the antimetabolite gemcitabine, which targets tumour cell progression in S-phase [24]. It is also worth noting that adding EdU, a weak antimetabolite, in combination with doxo, in BE(2)-C, resulted in a reduction in the number of newly formed colonies at day 10 from 8.3 ± 1.5 to 1.0 ± 1.0 compared to a single doxo treatment (data not shown). This indicates a specific vulnerability to antimetabolites when combined with doxo treatment in NB cell lines. A similar finding was previous reported where EdU had an anti-proliferating effect without being apparently cytotoxic [46]. Therefore alternative combination treatment with antimetabolites, at least during the first couple of rounds of therapy, could be a promising treatment option in therapy protocols in ultra high-risk and following relapsed NB where *TP53* mutations are more frequently occurring. Furthermore, these in vitro findings may have an impact on the development of new treatment strategies through that doxo today is commonly included in first-line and salvage therapy protocols. 

## 4. Materials and Methods

### 4.1. Cell Lines and Culture

BE(2)-C and Kelly were obtained from ATCC (Manassas, VA, USA). SK-N-AS, SK-N-FI and SK-N-DZ were a kind gift from professor Per Kogner at Karolinska Institutet. STR analysis (Eurofins Genomics, Ebersberg, Germany) confirmed the authenticity of the cell lines (data not shown). Basic characteristics of the NB cell lines are described in Table S1 [31,33,47]. Cells were cultured in RPMI 1640 medium supplemented with 10% fetal bovine serum, 2 mM L-glutamine and, 1% penicillin/streptomycin (all from Life Technologies Inc, Thermo Fisher Scientific, Stockholm, Sweden), at 37 °C, 5% CO_2_ with high humidity.

### 4.2. Treatment with Doxorubicin

Doxorubicin (doxo) (Apoteket AB, Stockholm, Sweden) was diluted in 1X PBS and administered to the culture medium. Doxo was added for 48 h at concentrations of 0.01 µM, 0.1 µM or 1 µM, to cells in logarithmic growth. For the repeated (1 + 1 µM) doxo treatment 1 µM doxo was added for 48 h followed by medium change and a second dose of 1 µM doxo for another 48 h. For mock treatment, 1X PBS was added to the culture medium.

### 4.3. Flow Cytometry

All five NB cell lines, in logarithmic growth, were treated with mock (1X PBS), or 0.01 µM, 0.1 µM, 1 µM doxo. Forty-eight hours post-treatment the cells were fixated with 70% EtOH before incubation with Hoechst 33343 (Applied Biosystems, Thermo Fisher Scientific, Stockholm, Sweden) according to the manufacturer’s protocol and run on a FACS Novocyte^®^ (Agilent, Santa Clara, CA, USA). The data were analysed and quantified by using NovoExpress^TM^ software (version 1.4, Agilent, Santa Clara, CA, USA). Each condition was run in triplicate. Statistics were performed on the proportion of cells within a specific gate.

### 4.4. Immunofluorescence

Following fixation for 15 min in ice-cold freshly prepared 4% paraformaldehyde (Merck, Molsheim, France), cells were incubated in TNB Buffer: 0.5g of blocking reagent (PerkinElmer, Stockholm, Sweden) to 100 mL TBS buffer (Tris/NaCl pH 7.4) for 30 min at room temperature. Cells were then incubated with the 1’ab diluted in 0.3% Triton X-100, 0.1% NaN_3_ in 1X PBS overnight at +4 °C. Following washing, cells were incubated with 2’ab diluted in TNB buffer, for 2 h at room temperature. The cells were washed and mounted with Prolong Gold antifade with DAPI (Thermo Fisher Scientific, Stockholm, Sweden) to visualize the nuclei. Stained cells were analysed using a Metafer^®^ Slide Scanning Platform (version 3.13.4, Metasystems, Heidelberg, Germany). A list of the antibodies and kits used is presented in Table S2.

### 4.5. Titration and MTS Assay

Cells, in logarithmic growth, were treated with a titration series of 10 to 0.01 μM ATM inhibitor (ATMi) (KU 60019, Tocris, Bristol, UK), CHK1 inhibitor (CHK1i) (PF-477736, Merck, Molsheim, France), CHK1/2 inhibitor (CHK1/2i) (AZD-7762, Merck, Molsheim, France), Wee1 (Wee1i) (Adavosertib, MedChemTronica, Stockholm, Sweden), or p21 (p21i) (UC2288, Merck, Molsheim, France) diluted in dimethyl sulfoxide (DMSO). After 72 h a 3-(4,5-dimethylthiazol-2-yl)-5-(3-carboxymethoxyphenyl)-2-(4-sulfophenyl)-2H-tetrazolium (MTS) assay was performed where 20 μL CellTiter 96^®^ AQueous One Solution Assay (Promega, Stockholm, Sweden) was added per 100 μL media. The plates were then incubated for 3 h and analysed in the microplate reader FLUOstar Omega (BMG LABTECH, Ortenberg, Germany). The absorbance at 490 nm and 690 nm was measured to assess the cell viability post-treatment. Each treatment was completed in triplicates. DMSO was used as a negative control.

### 4.6. Confluency Assay

Each cell line, in logarithmic growth, was treated at a concentration of 10 µM for ATMi and p21i or the corresponding IC50 value for each drug. The appropriate drugs either as a single agent ATMi, CHK1i, CHK1/2i, Wee1i, p21i, or as a combination with 1 µM doxo were added for 48 h followed by medium change. An amount of 1 μM doxo, 1X PBS (mock), or DMSO (mock) was used as negative controls. The plates were scanned by an IncuCyte S3 Live^®^ Cell Analysis System (Essen Bioscience, Welwyn Garden City, UK) every 48 h for 44 days. Fresh medium was added every 4 days. The proliferation was determined by measuring the cell confluency. Each treatment was done in five replicates and the data are presented as mean ± Standard Error of the Mean (SEM).

### 4.7. 5-Ethynyl-2′-Deoxyuridine

The thymidine analogue 5-Ethynyl-2′-deoxyuridine (EdU) (Applied Biosystems, Thermo Fisher Scientific, Stockholm, Sweden) was added at a concentration of 10 µM 48 h before fixation. EdU labelling was performed according to the manufacturer’s protocol.

### 4.8. Colony Formation

Kelly, SK-N-FI, and BE(2)-C cells, in logarithmic growth, were treated with 1 µM doxo in combination with 10 µM EdU for 48 h followed by medium change. Cells were allowed to grow, with fresh medium added every 4 days, until colonies were visible. The cells were fixed for 15 min in ice-cold freshly prepared 4% paraformaldehyde, EdU labelling was performed according to the manufacturer’s protocol. Cell density was visualised by DAPI staining and the number of EdU positive cells was deciphered as cells per scanned image (10× image) using a Metafer^®^ Slide Scanning Platform. Colony formation, with a threshold of >10 cells in proximity set for the definition of a colony, was assayed by ocular inspections.

### 4.9. Statistical Analysis

Data were analysed using Student’s *t*-test and two-way analysis of variance (ANOVA). Dunnett or Bonferroni post-hoc test was applied to adjust for multiple testing as indicated in the text. The software GraphPad Prism 7.03 (San Diego, CA, USA) was used for testing the normal distribution and generation of graphs. The immunofluorescence data were analysed in RStudio (version 1.2.5033, Boston, MA, USA) and the percentage of positive cells followed by the mean ± Standard Deviation (SD) were calculated for each combination.

## Figures and Tables

**Table 1:** Doxo-induced apoptosis (Sub-G1).

Cell Line	Mock	Doxo 0.01 µM	*p*	Doxo 0.1 µM	*p*	Doxo 1 µM	*p*
SK-N-DZ	5.3 ± 1.2	3.6 ± 1.1	*NS*	26 ± 2.5	****	18 ± 11	**
Kelly	0.9 ± 0.2	1.3 ± 0.9	*NS*	7.1 ± 1.4	*NS*	13 ± 7.3	**
SK-N-AS	0.5 ± 0.1	1.0 ± 0.4	*NS*	6.5 ± 1.0	*NS*	7.4 ± 2.3	*
SK-N-FI	11 ± 4.3	7.6 ± 2.7	*NS*	16 ± 4.6	*NS*	11 ± 2.5	*NS*
BE(2)-C	4.3 ± 0.9	3.9 ± 1.6	*NS*	12 ± 4.9	*NS*	6.3 ± 1.9	*NS*

Cultured cells were exposed to the indicated treatment (doxo/mock) and analysed after 48 h. Results are presented as percentage cells in Sub-G1. SK-N-DZ, Kelly, and SK-N-AS showed an increase in Sub-G1 after 0.1 µM and/or 1 µM doxo treatment. SK-N-FI and BE(2)-C showed no change in sub-G1 fractions. Mean ± SD of three experiments. * = *p* < 0.05, ** = *p* < 0.01, **** = *p* < 0.0001, *NS* = not significant *p* > 0.05. Two-way ANOVA and Dunnett post-hoc test.

**Table 2:** Analysis of cell cycle checkpoint proteins following doxo treatment for 48 h.

	pATM				pCHK1				pCHK2			
Cell Line	Mock		Doxo	*p*	Mock		Doxo	*p*	Mock		Doxo	*p*
SK-N-DZ	1.2 ± 0.3	<	89 ± 1.5	****	3.8 ± 1.9	≃	23 ± 17	*NS*	1.0 ± 0.5	<	6.2 ± 1.2	**
Kelly	3.1 ± 2.5	<	74 ± 2.2	****	0.5 ± 0.1	<	15 ± 5.2	**	0.6 ± 0.5	<	11 ± 4.0	**
SK-N-AS	1.4 ± 0.8	≃	3.1 ± 1.8	*NS*	0.6 ± 0.6	≃	1.6 ± 0.5	*NS*	0.5 ± 0.2	<	1.0 ± 0.6	*NS*
SK-N-FI	12 ± 5.5	<	82 ± 8.4	***	1.1 ± 0.4	≃	2.8 ± 2.1	*NS*	5.1 ± 2.5	<	33 ± 13	*
BE(2)-C	7.2 ± 3.9	<	86 ± 9.7	****	0.8 ± 0.5	<	11 ± 4.4	*	1.3 ± 0.4	<	6.4 ± 0.2	***

Cultured cells were exposed to the indicated treatment (doxo/mock) and analysed after 48 h. Results are presented as percentage cells positive for each marker. Robust expression of pATM was observed in all cell lines, except SK-N-AS. pCHK1 and pCHK2 showed varying increase in expression, however low, in the different cell lines, except SK-N-AS. Mean ± SD of 3-6 experiments. * = *p* < 0.05, ** = *p* < 0.01, *** = *p* < 0.001, **** = *p* < 0.0001, *NS* = not significant *p* > 0.05. Student’s *t*-test.

**Table 3:** Analysis of cell cycle checkpoint proteins following doxo treatment for 48 h.

	Wee1				p21				p27^Kip1^			
Cell Line	Mock		Doxo	*p*	Mock		Doxo	*p*	Mock		Doxo	*p*
SK-N-DZ	1.1 ± 0.4	<	9.5 ± 4.3	*	2.6 ± 1.0	<	29 ± 8.0	**	0.4 ± 0.3	≃	4.0 ± 3.2	*NS*
Kelly	0.2 ± 0.1	<	1.2 ± 0.4	*	18 ± 2.2	≃	23 ± 13	*NS*	0.1 ± 0.1	<	1.4 ± 0.3	**
SK-N-AS	0.1 ± 0.2	≃	0.2 ± 0.0	*NS*	2.5 ± 0.8	<	28 ± 3.0	***	0.0 ± 0.0	≃	0.1 ± 0.1	*NS*
SK-N-FI	1.1 ± 0.4	≃	1.6 ± 0.2	*NS*	23 ± 1.5	<	34 ± 3.9	**	2.3 ± 0.1	<	6.3 ± 1.1	**
BE(2)-C	2.1 ± 1.0	<	10 ± 3.8	*	2.0 ± 0.9	<	21 ± 1.6	****	0.7 ± 1.2	≃	0.2 ± 0.2	*NS*

Cultured cells were exposed to the indicated treatment (doxo/mock) and analysed after 48 h. Results are presented as percentage cells positive for each marker. Wee1, p21, and p27^Kip1^ showed varying increase in expression, however low, in the different cell lines. Mean ± SD of 3-6 experiments. * = *p* < 0.05, ** = *p* < 0.01, *** = *p* < 0.001, **** = *p* < 0.0001, *NS* = not significant *p* > 0.05. Student’s *t*-test.

**Table 4:** Marker analysis following single doxo treatment for 48 h.

	PH3+				EdU+				PH3+/EdU+				PH3+/EdU-			
Cell Line	Mock		Doxo	*p*	Mock		Doxo	*p*	Mock		Doxo	*p*	Mock		Doxo	*p*
SK-N-DZ	3.9 ± 1.4	<	11 ± 2.1	*	98 ± 0.7	>	81 ± 3.2	***	3.9 ± 1.4	<	7.7 ± 1.1	*	0.1 ± 0.1	<	2.9 ± 1.1	*
Kelly	5.8 ± 2.0	≃	3.6 ± 0.5	*NS*	92 ± 8.1	>	20 ± 17	**	5.5 ± 2.3	>	0.8 ± 0.8	**	0.3 ± 0.3	<	2.7 ± 0.4	*
SK-N-AS	1.2 ± 0.5	<	6.0 ± 2.4	*	93 ± 3.0	>	72 ± 6.3	***	1.2 ± 0.6	<	4.7 ± 2.0	*	0.1 ± 0.1	<	1.3 ± 0.7	*
SK-N-FI	4.9 ± 1.8	<	20 ± 5.0	**	65 ± 14	>	27 ± 1.5	**	3.8 ± 1.9	<	9.3 ± 2.8	*	1.1 ± 0.1	<	11 ± 2.2	**
BE(2)-C	6.0 ± 1.6	<	15 ± 1.5	****	98 ± 0.3	>	64 ± 9.1	****	6.0 ± 1.8	<	9.3 ± 0.9	**	0.0 ± 0.0	<	5.4 ± 0.9	****

Cultured cells were exposed to the treatment (doxo/mock) and analysed after 48 h. Results are presented as percentage cells positive for each marker. An increase in PH3 positive was observed in all cell lines, except Kelly. A reduction of EdU positive cells were observed in all tested cell lines. An increase in PH3+/EdU+ was observed in all cell lines, except Kelly, and an increase in PH3+/EdU- was observed in all cell lines. Mean ± SD of 3–6 experiments. * = *p* < 0.05, ** = *p* < 0.01, *** = *p* < 0.001, **** = *p* < 0.0001, NS= not significant *p* > 0.05. Student’s *t*-test

**Table 5:** Marker analysis following repeated doxo treatment for 48 + 48 h.

	PH3+				EdU+				PH3+/EdU+				PH3+/EdU-			
Cell line	Mock		Doxo	*p*	Mock		Doxo	*p*	Mock		Doxo	*p*	Mock		Doxo	*p*
SK-N-DZ	1.8 ± 1.2	<	7.8 ± 2.8	*	99 ± 0.4	>	14 ± 8.0	****	1.8 ± 1.2	≃	0.8 ± 0.7	*NS*	0.0 ± 0.0	<	6.9 ± 2.1	**
Kelly	3.2 ± 0.7	<	24 ± 6.0	**	98 ± 1.5	>	0.0 ± 0.0	****	3.2 ± 0.7	>	0.0 ± 0.0	**	0.0 ± 0.0	<	24 ± 6.0	**
SK-N-AS	3.7 ± 1.8	≃	4.6 ± 1.4	*NS*	97 ± 1.2	>	12 ± 2.0	****	3.6 ± 1.8	>	1.2 ± 0.7	*	0.1 ± 0.1	<	3.5 ± 0.8	***
SK-N-FI	3.0 ± 0.1	<	15 ± 0.5	***	76 ± 2.9	>	7.1 ± 3.2	****	2.9 ± 0.2	≃	3.5 ± 1.2	*NS*	0.1 ± 0.1	<	11 ± 0.2	****
BE(2)-C	8.4 ± 4.7	<	35 ± 4.8	**	98 ± 0.4	>	5.5 ± 0.7	****	8.4 ± 4.7	≃	1.3 ± 0.6	*NS*	0.0 ± 0.1	<	34 ± 4.2	****

Cultured cells were exposed to the treatment (doxo/mock) and analysed after 48 + 48 h. Results are presented as percentage cells positive for each marker. An increase in PH3 positive was observed in all cell lines, except SK-N-AS. A reduction in EdU positive cells was observed in all cell lines. A small population of PH3+/EdU+ was observed in all cell lines except Kelly, and an increase in PH3+/EdU- was observed in all cell lines. Mean ± SD of 3–6 experiments. * = *p* < 0.05, ** = *p* < 0.01, *** = *p* < 0.001, **** = *p* < 0.0001, *NS* = not significant *p* > 0.05. Student’s *t*-test.

## Data Availability

The data that support the findings of this study are available from the corresponding author upon request.

## Supplementary Materials

The following are available online at https://www.mdpi.com/article/10.3390/ijms22073664/s1, Table S1: NB cell lines tested, Table S2: Antibodies and kits used for immunfluorescence, Table S3: Flow cytometry analysis of *TP53* mut NB cell lines following mock or doxo treatment, Table S4: Analysis of cell cycle checkpoint proteins in BE(2)-C cells following repeated doxo treatment for 48 + 48 h, Table S5: Heterogeneities in cell cycle checkpoint activation following 1 µM doxo in *TP53* mut NB cell lines.

## References

[1] Brodeur G.M. (2003). Neuroblastoma: Biological insights into a clinical enigma. Nat. Rev. Cancer.

[2] Viprey V.F., Gregory W.M., Corrias M.V., Tchirkov A., Swerts K., Vicha A., Dallorso S., Brock P., Luksch R., Valteau-Couanet D. (2014). Neuroblastoma mRNAs Predict Outcome in Children With Stage 4 Neuroblastoma: A European HR-NBL1/SIOPEN Study. J. Clin. Oncol..

[3] Bedard P.L., Hansen A.R., Ratain M.J., Siu L.L. (2013). Tumour heterogeneity in the clinic. Nat. Cell Biol..

[4] Seeger R.C., Reynolds C.P. (1991). Treatment of High-Risk Solid Tumors of Childhood with Intensive Therapy and Autologous Bone Marrow Transplantation. Pediatr. Clin. N. Am..

[5] Pinto N.R., Applebaum M.A., Volchenboum S.L., Matthay K.K., London W.B., Ambros P.F., Nakagawara A., Berthold F., Schleiermacher G., Park J.R. (2015). Advances in Risk Classification and Treatment Strategies for Neuroblastoma. J. Clin. Oncol..

[6] Pastor E.R., Mousa S.A. (2019). Current management of neuroblastoma and future direction. Crit. Rev. Oncol..

[7] Alexander N., Marrano P., Thorner P., Naranjo A., Van Ryn C., Martinez D., Batra V., Zhang L., Irwin M.S., Baruchel S. (2019). Prevalence and Clinical Correlations of Somatostatin Receptor-2 (SSTR2) Expression in Neuroblastoma. J. Pediatr. Hematol..

[8] Keshelava N., Seeger R.C., Groshen S., Reynolds C.P. (1998). Drug resistance patterns of human neuroblastoma cell lines derived from patients at different phases of therapy. Cancer Res..

[9] Gewirtz D. (1999). A critical evaluation of the mechanisms of action proposed for the antitumor effects of the anthracycline antibiotics adriamycin and daunorubicin. Biochem. Pharmacol..

[10] Harper J.W., Elledge S.J. (2007). The DNA Damage Response: Ten Years After. Mol. Cell.

[11] Vousden K.H., Lu X. (2002). Live or let die: The cell’s response to p53. Nat. Rev. Cancer.

[12] Kastan M.B., Bartek J. (2004). Cell-cycle checkpoints and cancer. Nature.

[13] Aliouat-Denis C.-M., Dendouga N., Wyngaert I.V.D., Goehlmann H., Steller U., Van De Weyer I., Van Slycken N., Andries L., Kass S., Luyten W. (2005). p53-Independent Regulation of p21Waf1/Cip1 Expression and Senescence by Chk2. Mol. Cancer Res..

[14] Lee J., Kumagai A., Dunphy W.G. (2001). Positive Regulation of Wee1 by Chk1 and 14-3-3 Proteins. Mol. Biol. Cell.

[15] Abraham R.T. (2001). Cell cycle checkpoint signaling through the ATM and ATR kinases. Genes Dev..

[16] Abukhdeir A.M., Park B.H. (2008). p21 and p27: Roles in carcinogenesis and drug resistance. Expert Rev. Mol. Med..

[17] Curtin N.J. (2012). DNA repair dysregulation from cancer driver to therapeutic target. Nat. Rev. Cancer.

[18] Cole K.A., Maris J.M. (2012). New Strategies in Refractory and Recurrent Neuroblastoma: Translational Opportunities to Impact Patient Outcome. Clin. Cancer Res..

[19] Gu L., Chu P., Lingeman R., McDaniel H., Kechichian S., Hickey R.J., Liu Z., Yuan Y.-C., Sandoval J.A., Fields G.B. (2015). The Mechanism by Which MYCN Amplification Confers an Enhanced Sensitivity to a PCNA-Derived Cell Permeable Peptide in Neuroblastoma Cells. EBioMedicine.

[20] Mlakar V., Mlakar S.J., Lopez G., Maris J.M., Ansari M., Gumy-Pause F. (2017). 11q deletion in neuroblastoma: A review of biological and clinical implications. Mol. Cancer.

[21] Hosoi G., Hara J., Okamura T., Osugi Y., Ishihara S., Fukuzawa M., Okada A., Okada S., Tawa A. (1994). Low frequency of the p53 gene mutations in neuroblastoma. Cancer.

[22] Carr-Wilkinson J., O’Toole K., Wood K.M., Challen C.C., Baker A.G., Board J.R., Evans L., Cole M., Cheung N.K., Boos J. (2010). High Frequency of p53/MDM2/p14ARF Pathway Abnormalities in Relapsed Neuroblastoma. Clin. Cancer Res..

[23] Carr-Wilkinson J., Griffiths R., Elston R., Gamble L.D., Goranov B., Redfern C., Lunec J., Tweddle D.A. (2011). Outcome of the p53-mediated DNA damage response in neuroblastoma is determined by morphological subtype and MYCN expression. Cell Cycle.

[24] Xu H., Cheung I.Y., Wei X.X., Tran H., Gao X., Cheung N.-K.V. (2011). Checkpoint kinase inhibitor synergizes with DNA-damaging agents in G1checkpoint-defective neuroblastoma. Int. J. Cancer.

[25] Fulda S., Honer M., Menke-Moellers I., Berthold F. (1995). Antiproliferative potential of cytostatic drugs on neuroblastoma cells in vitro. Eur. J. Cancer.

[26] Barpe D.R., Rosa D.D., Froehlich P.E. (2010). Pharmacokinetic evaluation of doxorubicin plasma levels in normal and overweight patients with breast cancer and simulation of dose adjustment by different indexes of body mass. Eur. J. Pharm. Sci..

[27] Hendzel M.J., Wei Y., Mancini M.A., Van Hooser A., Ranalli T., Brinkley B.R., Bazett-Jones D.P., Allis C.D. (1997). Mitosis-specific phosphorylation of histone H3 initiates primarily within pericentromeric heterochromatin during G2 and spreads in an ordered fashion coincident with mitotic chromosome condensation. Chromosoma.

[28] Visconti R., Della Monica R., Grieco D. (2016). Cell cycle checkpoint in cancer: A therapeutically targetable double-edged sword. J. Exp. Clin. Cancer Res..

[29] Kastan M.B., Onyekwere O., Sidransky D., Vogelstein B., Craig R.W. (1991). Participation of p53 protein in the cellular response to DNA damage. Cancer Res..

[30] Hultman I., Haeggblom L., Rognmo I., Edqvist J.J., Blomberg E., Ali R., Phillips L., Sandstedt B., Kogner P., Fard S.S. (2018). Doxorubicin-provoked increase of mitotic activity and concomitant drain of G0-pool in therapy-resistant BE(2)-C neuroblastoma. PLoS ONE.

[31] Cole K.A., Huggins J., Laquaglia M., Hulderman C.E., Russell M.R., Bosse K., Diskin S.J., Attiyeh E.F., Sennett R., Norris G. (2011). RNAi screen of the protein kinome identifies checkpoint kinase 1 (CHK1) as a therapeutic target in neuroblastoma. Proc. Natl. Acad. Sci. USA.

[32] Ando K., Nakamura Y., Nagase H., Nakagawara A., Koshinaga T., Wada S., Makishima M. (2019). Co-Inhibition of the DNA Damage Response and CHK1 Enhances Apoptosis of Neuroblastoma Cells. Int. J. Mol. Sci..

[33] Ribelles A.J., Barberá S., Yáñez Y., Gargallo P., Segura V., Juan B., Noguera R., Piqueras M., Fornés-Ferrer V., De Mora J.F. (2019). Clinical Features of Neuroblastoma with 11q Deletion: An Increase in Relapse Probabilities in Localized and 4S Stages. Sci. Rep..

[34] Maya-Mendoza A., Moudry P., Merchut-Maya J.M., Lee M., Strauss R., Bartek J. (2018). High speed of fork progression induces DNA replication stress and genomic instability. Nat. Cell Biol..

[35] Ronson G.E., Piberger A.L., Higgs M.R., Olsen A.L., Stewart G.S., McHugh P.J., Petermann E., Lakin N.D. (2018). PARP1 and PARP2 stabilise replication forks at base excision repair intermediates through Fbh1-dependent Rad51 regulation. Nat. Commun..

[36] Sanmartín E., Muñoz L., Piqueras M., Sirerol J.A., Berlanga P., Cañete A., Castel V., De Mora J.F. (2017). Deletion of 11q in Neuroblastomas Drives Sensitivity to PARP Inhibition. Clin. Cancer Res..

[37] Hsu C.-H., Altschuler S.J., Wu L.F. (2019). Patterns of Early p21 Dynamics Determine Proliferation-Senescence Cell Fate after Chemotherapy. Cell.

[38] Russell M.R., Levin K., Rader J., Belcastro L., Li Y., Martinez D., Pawel B., Shumway S.D., Maris J.M., Cole K.A. (2013). Combination Therapy Targeting the Chk1 and Wee1 Kinases Shows Therapeutic Efficacy in Neuroblastoma. Cancer Res..

[39] Hanmod S.S., Wang G., Edwards H., Buck S.A., Ge Y., Taub J.W., Wang Z. (2014). Targeting histone deacetylases (HDACs) and Wee1 for treating high-risk neuroblastoma. Pediatr. Blood Cancer.

[40] Southgate H.E.D., Chen L., Curtin N.J., Tweddle D.A. (2020). Targeting the DNA Damage Response for the Treatment of High Risk Neuroblastoma. Front. Oncol..

[41] Dalton W.B., Yang V.W. (2009). Role of prolonged mitotic checkpoint activation in the formation and treatment of cancer. Futur. Oncol..

[42] Syljuåsen R.G. (2007). Checkpoint adaptation in human cells. Oncogene.

[43] Swift L.H., Golsteyn R.M. (2016). Cytotoxic amounts of cisplatin induce either checkpoint adaptation or apoptosis in a concentration-dependent manner in cancer cells. Biol. Cell.

[44] Kubara P.M., Kernéis-Golsteyn S., Studény A., Lanser B.B., Meijer L., Golsteyn R.M. (2012). Human cells enter mitosis with damaged DNA after treatment with pharmacological concentrations of genotoxic agents. Biochem. J..

[45] Shaltiel I.A., Krenning L., Bruinsma W., Medema R.H. (2015). The same, only different—DNA damage checkpoints and their reversal throughout the cell cycle. J. Cell Sci..

[46] Ross H.H., Rahman M., Levkoff L.H., Millette S., Martin-Carreras T., Dunbar E.M., Reynolds B.A., Laywell E.D. (2011). Ethynyldeoxyuridine (EdU) suppresses in vitro population expansion and in vivo tumor progression of human glioblastoma cells. J. Neuro-Oncol..

[47] Harenza J.L., Diamond M.A., Adams R.N., Song M.M., Davidson H.L., Hart L.S., Dent M.H., Fortina P., Reynolds C.P., Maris J.M. (2017). Transcriptomic profiling of 39 commonly-used neuroblastoma cell lines. Sci. Data.

