# Supplementary material for: Heterogeneities in Cell Cycle Checkpoint Activation Following Doxorubicin Treatment Reveal Targetable Vulnerabilities in TP53 Mutated Ultra High-Risk Neuroblastoma Cell Lines
Source: Int J Mol Sci. 2021 Apr 1;22(7):3664. doi: 10.3390/ijms22073664 (PMC8036447; doi:10.3390/ijms22073664)
Supplement: Supplementary file 1 [file ijms-22-03664-s001.zip › Supplementary Files/Table S1.docx]

Table S1: NB cell lines tested.

| **Cell line** | ***TP53***  ***status*** | ***NMYC status*** | ***11q***  ***status*** | ***17q***  ***status*** | ***Alk***  ***Status*** | **Origin** |
| --- | --- | --- | --- | --- | --- | --- |
| SK-N-DZ | mutated | amplified  (transcriptionally upregulates CHK1) | deletion (q21-qter) | gain | wild type | Metastasis location: Bone marrow  Patient age: 2 years  Gender: Girl |
| Kelly | mutated | amplified  (transcriptionally upregulates CHK1) | deletion (q23.3-qter) | gain | mutated  F1174L | Metastasis location: brain  Patient age: 1 year  Gender: Girl |
| SK-N-AS | mutated | wild type | deletion (q13.4-qter;  impaired ATM and CHK1) | gain | wild type | Metastasis location: Bone marrow  Patient age: 6 years  Gender: Girl |
| SK-N-FI | mutated | wild type | wild type | gain | wild type | Metastasis location: Bone marrow  Patient age: 11 years  Gender: Boy |
| BE(2)-C | mutated | amplified  (transcriptionally upregulates CHK1) | wild type | gain | wild type | Metastasis location: Bone marrow  Patient age: 22 months  Gender: Boy  Additional info: Cloned subline of SK-N-BE(2). Retrieved after repeated chemo and radiotherapy. |
