# Supplementary material for: Heterogeneities in Cell Cycle Checkpoint Activation Following Doxorubicin Treatment Reveal Targetable Vulnerabilities in TP53 Mutated Ultra High-Risk Neuroblastoma Cell Lines
Source: Int J Mol Sci. 2021 Apr 1;22(7):3664. doi: 10.3390/ijms22073664 (PMC8036447; doi:10.3390/ijms22073664)
Supplement: Supplementary file 1 [file ijms-22-03664-s001.zip › Supplementary Files/Table S2.docx]

Table S2. Antibodies and kits used for immunocytochemistry.

| **Antigen** | **Source** | **Dilution** | **2^nd^ detection** |
| --- | --- | --- | --- |
| pATM | Abcam ab36810, mouse anti-ATM (phosphor S1981) | 1:500 | Goat anti-mouse 1:200 |
| pCHK1 | ThermoFisher PA5-104213, rabbit anti-CHK1 (phosphor S296) | 1:500 | Donkey anti-rabbit Cy3.5 1:800 |
| pCHK2 | Invitrogen PA578361, rabbit anti-CHK2 (phosphor T68) | 1:500 | Donkey anti- rabbit Cy3.5 1:800 |
| Wee1 | Cell Signaling #13084S, rabbit anti-Wee1 | 1:300 | Donkey anti-rabbit Cy3.5 1:300 |
| p21^Waf1/Cip1^ | Cell Signaling #2947S rabbit anti-p21 (12D1) | 1:1000 | Donkey  anti-rabbit Cy3.5 1:800 |
| p27^Kip1^ | Cell signaling #83630S mouse anti-p27 (SX53G8.5) | 1:250 | Goat anti-  mouse Cy2 1:200 |
| PH3 | Merck Cat: 06-570, rabbit anti-phospho-Histone H3 | 1:4000 | Goat anti-rabbit AF488 1:1000 |
| EdU | Click-it EdUAlexa Fluor 594 Imaging Kit (C10339, Invitrogen) | According to manufacturer’s protocol | NA |
