# Supplementary material for: Heterogeneities in Cell Cycle Checkpoint Activation Following Doxorubicin Treatment Reveal Targetable Vulnerabilities in TP53 Mutated Ultra High-Risk Neuroblastoma Cell Lines
Source: Int J Mol Sci. 2021 Apr 1;22(7):3664. doi: 10.3390/ijms22073664 (PMC8036447; doi:10.3390/ijms22073664)
Supplement: Supplementary file 1 [file ijms-22-03664-s001.zip › Supplementary Files/Table S3.docx]

Table S3: Flow cytometry analysis of *TP53* mut NB cell lines following mock or doxo treatment.

| **SK-N-DZ** | **Mock** | **Doxo**  **0.01 µM** | **Doxo**  **0.1 µM** | **Doxo**  **1 µM** |
| --- | --- | --- | --- | --- |
| **2N**  (G0/G1) | 55 ± 10 | 52 ± 4.1  NS | 16 ± 5.1  **** | 8.0 ± 3.3  **** |
| **S** | 28 ± 8.1 | 30 ± 0.7  NS | 25 ± 5.5  NS | 39 ± 3.4  * |
| **4N**  (G2/M) | 11 ± 1.5 | 14 ± 2.4  NS | 32 ± 12  **** | 35 ± 11  **** |
| **>4n** | 0.8 ± 0.3 | 0.7 ± 0.3  NS | 0.8 ± 0.2  NS | 0.9 ± 0.3  NS |
| **Kelly** | **Mock** | **Doxo**  **0.01 µM** | **Doxo**  **0.1 µM** | **Doxo**  **1 µM** |
| **2N**  (G0/G1) | 64 ± 7.3 | 63 ± 8.9  NS | 41 ± 5.9  **** | 11 ± 4.1  **** |
| **S** | 28 ± 3.4 | 29 ± 3.9  NS | 18 ± 2.7  * | 69 ± 3.9  **** |
| **4N**  (G2/M) | 6.6 ± 4.0 | 7.2 ± 5.5  NS | 33 ± 2.8  **** | 5.8 ± 4.3  NS |
| **>4n** | 0.6 ± 0.4 | 0.4 ± 0.4  NS | 0.8 ± 0.2  NS | 0.9 ± 0.1  NS |
| **SK-N-AS** | **Mock** | **Doxo**  **0.01 µM** | **Doxo**  **0.1 µM** | **Doxo**  **1 µM** |
| **2N**  (G0/G1) | 51 ± 1.2 | 46 ± 6.4  NS | 7.2 ± 3.6  **** | 8.8 ± 2.4  **** |
| **S** | 32 ± 2.3 | 29 ± 6.4  NS | 18 ± 3.0  **** | 77 ± 4.4  **** |
| **4N**  (G2/M) | 15 ± 1.9 | 24 ± 0.6  ** | 66 ± 5.7  **** | 5.4 ± 3.5  ** |
| **>4n** | 1.0 ± 0.1 | 0.8 ± 0.1  NS | 2.0 ± 0.5  NS | 1.4 ± 0.9  NS |
| **SK-N-FI** | **Mock** | **Doxo**  **0.01 µM** | **Doxo**  **0.1 µM** | **Doxo**  **1 µM** |
| **2N**  (G0/G1) | 66 ± 8.7 | 66 ± 2.2  NS | 37 ± 3.3  **** | 26 ± 6.3  **** |
| **S** | 16 ± 3.1 | 18 ± 1.1  NS | 20 ± 4.1  NS | 30 ± 4.4  *** |
| **4N**  (G2/M) | 6.6 ± 2.1 | 8.4 ± 1.5  NS | 27 ± 5.1  **** | 31 ± 7.2  **** |
| **>4n** | 0.6 ± 0.6 | 0.4 ± 0.1  NS | 1.2 ± 0.4  NS | 1.5 ± 0.3  NS |
| **BE(2)-C** | **Mock** | **Doxo**  **0.01 µM** | **Doxo**  **0.1 µM** | **Doxo**  **1 µM** |
| **2N**  (G0/G1) | 62 ± 4.8 | 63 ± 2.8  NS | 13 ± 11  **** | 8.8 ± 3.7  **** |
| **S** | 23 ± 1.9 | 21 ± 1.2  NS | 22 ± 13  NS | 33 ± 6.5  NS |
| **4N**  (G2/M) | 10 ± 2.4 | 11 ± 0.9  NS | 52 ± 27  **** | 49 ± 6.2  **** |
| **>4n** | 1.3 ± 1.0 | 1.1 ± 0.4  NS | 1.0 ± 0.4  NS | 2.2 ± 0.9  NS |
| Cultured cells were exposed to the indicated treatment (doxo / mock) and, analysed after 48 hours. Results are presented as percentage cells in each cell cycle phase. All tested cell lines showed a dose dependent reduction in G0/G1- together with accumulation in S- and/or G2/M-phases. Mean ± SD of 3 experiments. * = p<0.05, **= p<0.01, **** = p<0.0001, NS= not significant p>0.05. Two-way ANOVA and Dunnett post-hoc test. | | | | |
