# Supplementary material for: Heterogeneities in Cell Cycle Checkpoint Activation Following Doxorubicin Treatment Reveal Targetable Vulnerabilities in TP53 Mutated Ultra High-Risk Neuroblastoma Cell Lines
Source: Int J Mol Sci. 2021 Apr 1;22(7):3664. doi: 10.3390/ijms22073664 (PMC8036447; doi:10.3390/ijms22073664)
Supplement: Supplementary file 1 [file ijms-22-03664-s001.zip › Supplementary Files/Table S4.docx]

Table S4: Analysis of cell cycle checkpoint proteins in BE(2)-C cells following repeated doxo treatment for 48+48 hours

| **BE(2)-C** | **Mock** |  | **Doxo**  **1+1 µM** | ***p*** |
| --- | --- | --- | --- | --- |
| pATM | 1.3 ± 0.6 | < | 90 ± 13 | ****** |
| pCHK1 | 2.3 ± 1.8 | *≃* | 4.3 ± 1.5 | *NS* |
| pCHK2 | 0.1 ± 0.2 | *≃* | 0.2 ± 0.1 | *NS* |
| Wee1 | 8.4 ± 2.8 | < | 17 ± 1.7 | **** |
| p21 | 1.7 ± 0.7 | < | 39 ± 2.8 | ****** |
| p27^Kip1^ | 0.0 ± 0.0 | < | 2.8 ± 0.4 | **** |
| Cultured cells were exposed to the indicated treatment (doxo / mock) and analysed after 48 hours. Results are presented as percentage cells positive for each marker. An increase in pATM positive cells was observed. Number of pCHK1 or pCHK2 positive cells following treatment was similar as the mock treatment. Instead increased fraction of positive cells was observed for Wee1, p21, and p27^Kip1^ following treatment. Mean ± SD of 3-6 experiments. **= p<0.01, **** = p<0.0001, NS= not significant p>0.05. Student´s t-test. | | | | |
