# Supplementary material for: Heterogeneities in Cell Cycle Checkpoint Activation Following Doxorubicin Treatment Reveal Targetable Vulnerabilities in TP53 Mutated Ultra High-Risk Neuroblastoma Cell Lines
Source: Int J Mol Sci. 2021 Apr 1;22(7):3664. doi: 10.3390/ijms22073664 (PMC8036447; doi:10.3390/ijms22073664)
Supplement: Supplementary file 1 [file ijms-22-03664-s001.zip › Supplementary Files/Table S5.docx]

Table S5: Heterogeneities in cell cycle checkpoint activation following 1 µM doxo in *TP53* mut NB cell lines.

|  | **Flow cytometry** | **IF** | **IncuCyte** | **IF** | |
| --- | --- | --- | --- | --- | --- |
| **Cell line** | **Increase** | **Increase** | **Delay in growth -**  **combination** | **Single treatment**  **Increase** | **Double treatment**  **Increase** |
| SK-N-DZ | Sub-G1,  S- and G2/M-phase | pATM, pCHK2, Wee1, p21 | ATMi | PH3+/EdU+, PH3+/EdU- | PH3+/EdU- |
| Kelly | Sub-G1,  S-phase | pATM, pCHK1, pCHK2, Wee1, p27Kip1 | p21i | PH3+/EdU- | PH3+/EdU- |
| SK-N-AS | Sub-G1, S-phase | p21 | NS | PH3+/EdU+, PH3+/EdU- | PH3+/EdU- |
| SK-N-FI | S- and G2/M-phase | pATM, pCHK2, p21, p27Kip1 | Wee1i  (initial delay) | PH3+/EdU+, PH3+/EdU- | PH3+/EdU- |
| BE(2)-C | G2/M-phase | pATM, pCHK1, pCHK2, Wee1, p21 | CHK1, Wee1i, p21i | PH3+/EdU+, PH3+/EdU- | PH3+/EdU- |
| IF: Immunofluorescence, NS: No significant | | | | | |
